# Novel Botulinum Toxin Injection Protocols for Parkinson Tremor and Essential Tremor – the Yale Technique and Sensor-Based Kinematics Procedure for Safe and Effective Treatment

**DOI:** 10.5334/tohm.582

**Published:** 2020-12-31

**Authors:** Shivam Om Mittal, Mandar Jog, Jack Lee, Bahman Jabbari

**Affiliations:** 1 Movement Disorders, Neurological Institute, Cleveland Clinic Abu Dhabi, AE; 2 London Movement Disorders Center, London, Ontario, CA; 3 Yale University, New Haven, CT, US

**Keywords:** Botulinum toxin, Botulinum neurotoxin, onabotulinumtoxinA, incobotulinumtoxinA, Essential tremor, Parkinson disease tremor, Tremor

## Abstract

**Background:**

Hand tremor associated with Parkinson disease (PD) and essential tremor (ET) can often become challenging to treat in clinical practice. Local injections of botulinum toxin-A (BoNT-A) for hand tremor is an evolving field with newer injection techniques being utilized in clinical studies. The utility of BoNT-A therapy for ET and PD-tremor however, has been questioned based on the high incidence of finger and hand weakness after treatment.

**Method:**

The study includes detailed analysis of the techniques utilized in BoNT injection in ET and PD tremor.

**Results:**

There were 4 high-quality investigations which consisted of Class I or II double-blind placebo-controlled trials and one medium-quality study that was a prospective, open label, class III investigation.

**Discussion:**

This paper discusses two recently developed technology-based injection methods for BoNT-A therapy of ET and PD tremor, which includes comprehensive EMG screening of forearm and arm muscles with selective injections (Yale method) and the whole arm kinematic tremor assessment developed by Jog et al. In recent years, controlled, blinded studies of these two methods have shown significant post-injection reduction of finger, hand and whole limb tremor compared to the previously published controlled clinical trials not using these methodologies.

## Introduction

Tremor is described as involuntary rhythmic oscillations due to muscular contractions [1]. There are several types of tremor and two of the most common include those associated with Parkinson’s disease (PD) and essential tremor (ET) [1,2]. Approximately 60–70% of PD patients presents with tremor. Classical tremor in PD is rest tremor involving unilateral upper limb starting as a pill rolling tremor and can eventually progress to bilateral upper limbs, lower limbs or chin/jaw [3]. Most of the PD patients have excellent control of tremor with the conventional medical treatment such as levodopa therapy, dopamine agonist, and anticholinergics. However, 30% of PD tremor patients are refractory to dopamine treatment and the tremor does not improve even with higher dose of dopaminergic medications. These patients are exposed to high doses of dopaminergic medications which pre-disposes them to the side-effects and motor fluctuations. Deep Brain Stimulation is possible option for these patients, but this is a surgical treatment which has strict inclusion criteria and has its own risk of complications [3,4].

Essential tremor usually presents with 4 to 10 Hz, action tremor including both postural and kinetic tremor, typically affects both upper limbs. The description of ET is somewhat ambiguous and has therefore compelled the development of consensus criteria to provide correct diagnosis of this disorder. According to the Task Force on Tremor of the International Parkinson and Movement Disorder Society, there are currently two types of essential tremor, which include ET and ET plus. In ET there should be: isolated tremor syndrome identified by bilateral upper limb action tremor, a duration of at least 3 years, “absence of other neurological signs, such as dystonia, ataxia, or parkinsonism” and it may or may not be accompanied by “tremor in other locations (e.g., head, voice, or lower limbs)”[2].

Individuals with severe tremor can experience functional disability, highlighting the need for effective therapeutic strategies [1,2]. Various treatment methods have been developed for the management of tremor including deep brain stimulation (DBS), MRI-guided focused ultrasound, and pharmacotherapy [5]; each having their own disadvantages. Betablockers and anticonvulsants fail in 30% of ET patients and their side-effects result in discontinuation of treatment in another 25–30% of individuals [6,7]. Despite the efficacy of thalamic, subthalamic nuclei and globus pallidus interna DBS, there is a 3–4% risk of adverse events like intracerebral hemorrhage, in addition to the fact that it is a surgical procedure which is not well-accepted by most patients [8]. MRI guided focused ultrasound (MRgFUS) is an incisionless surgery causing thalamotomy with focused ultrasound rays. The safety and long-term effects of the procedure are still unclear. Temporary side effects are very common which improves gradually but up to 15% patients were reported to have permanent deficits after thalamotomy such as sensory symptoms, weakness and gait unsteadiness. The outcome of MRgFUS procedures have been variable as well, tremor reduction ranged from –20% to +88% in the largest RCT performed so far. The tremor control in these patients can worsen by 23% over the first year [9,10].

Botulinum neurotoxins (BoNTs) are proteins derived from the bacterium *Clostridium botulinum* that have been used as a drug in clinical practice. Types A and B of this toxin are being widely used for the treatment of various movement disorders with a relatively high success rate, as indicated by clinical trials and practical experience. Its main mechanism of action is by preventing the release of acetylcholine from presynaptic vesicles via deactivating pre-synaptic SNARE proteins [11,12].

The number of high-quality studies focusing on BoNT as a therapeutic option for managing tremor, is limited [13,14,15,16] (Figure 2). Earlier publications have questioned the application of this toxin for the treatment of limb tremor, due to the high incidence of hand and finger weakness following injections [13,14]. However, use of more current methods suggested in the past 3 years such as the Yale protocol and standardized kinematic tremor assessment of the whole arm have significantly reduced this side effect in patients with ET and PD tremor while aiming to have the individual regain their hand and arm function [15,16,17].

## Methods

### Research and Results

**Figure 1:**
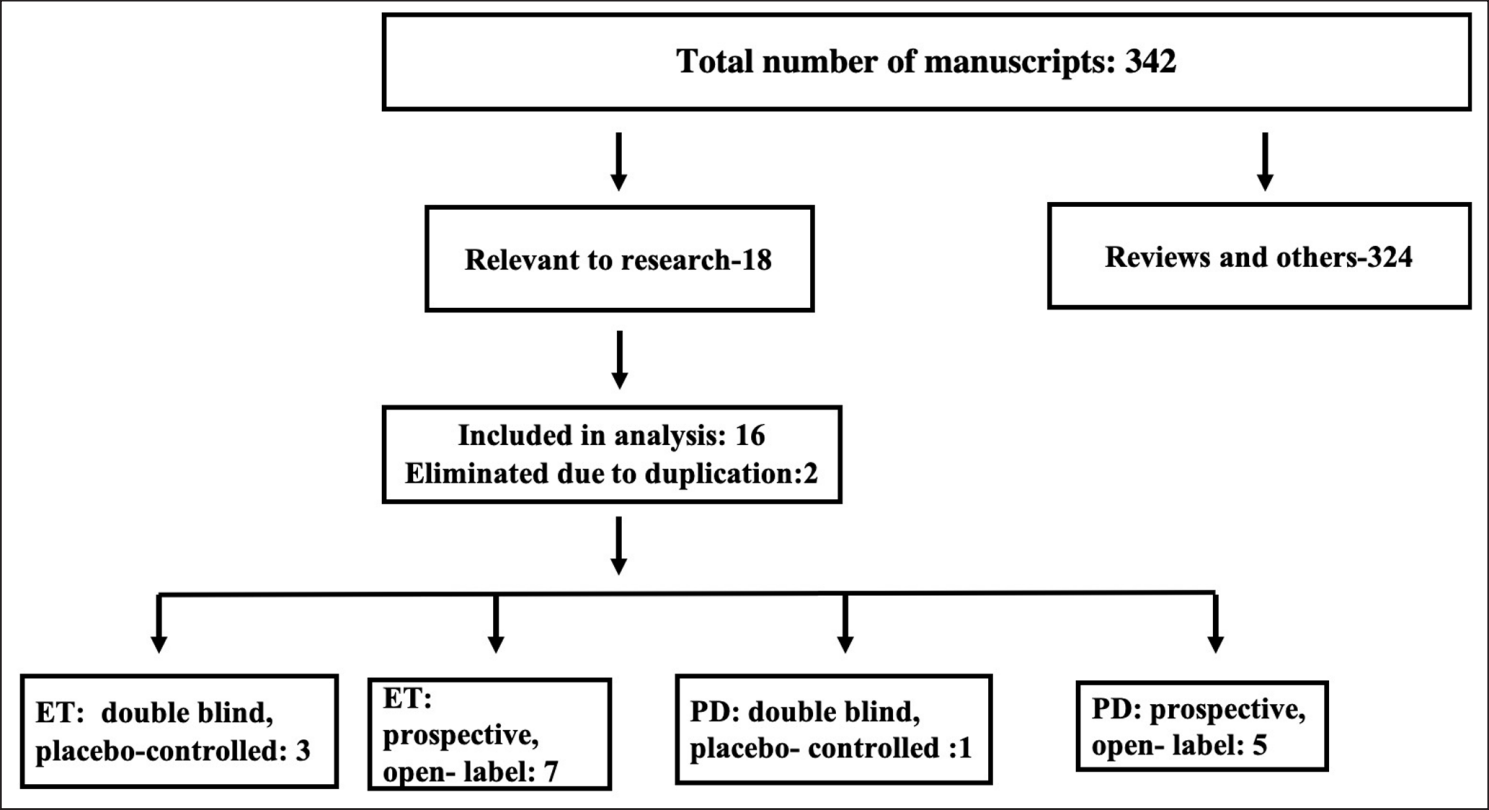
Review of Literature and the studies included.

The purpose of this study was to identify novel objective techniques through which botulinum toxin injections reduced ET or PD tremor effectively yet caused less weakness compare with the previously reported methodology. An electronic search was performed on Medline using “Tremor”, “Essential Tremor” and “Parkinson disease tremor” as keywords. Each of these, were crossed with “Botulinum Toxin OR Botulinum Neurotoxin” (Figure 1). As of January 1st, 2020, there were a total of 342 articles, of which 18 were relevant and included in the review. These publications were classified according to the criteria of the American Academy of Neurology [18,19] shown in Table 1. There were 4 high-quality investigations which consisted of Class I or II double-blind placebo-controlled trials and one medium-quality study that was a prospective, open label, class III investigation [15,16,17]. These five publications report a novel injection method, which will be the main topic under discussion in this review.

**Figure 2:**
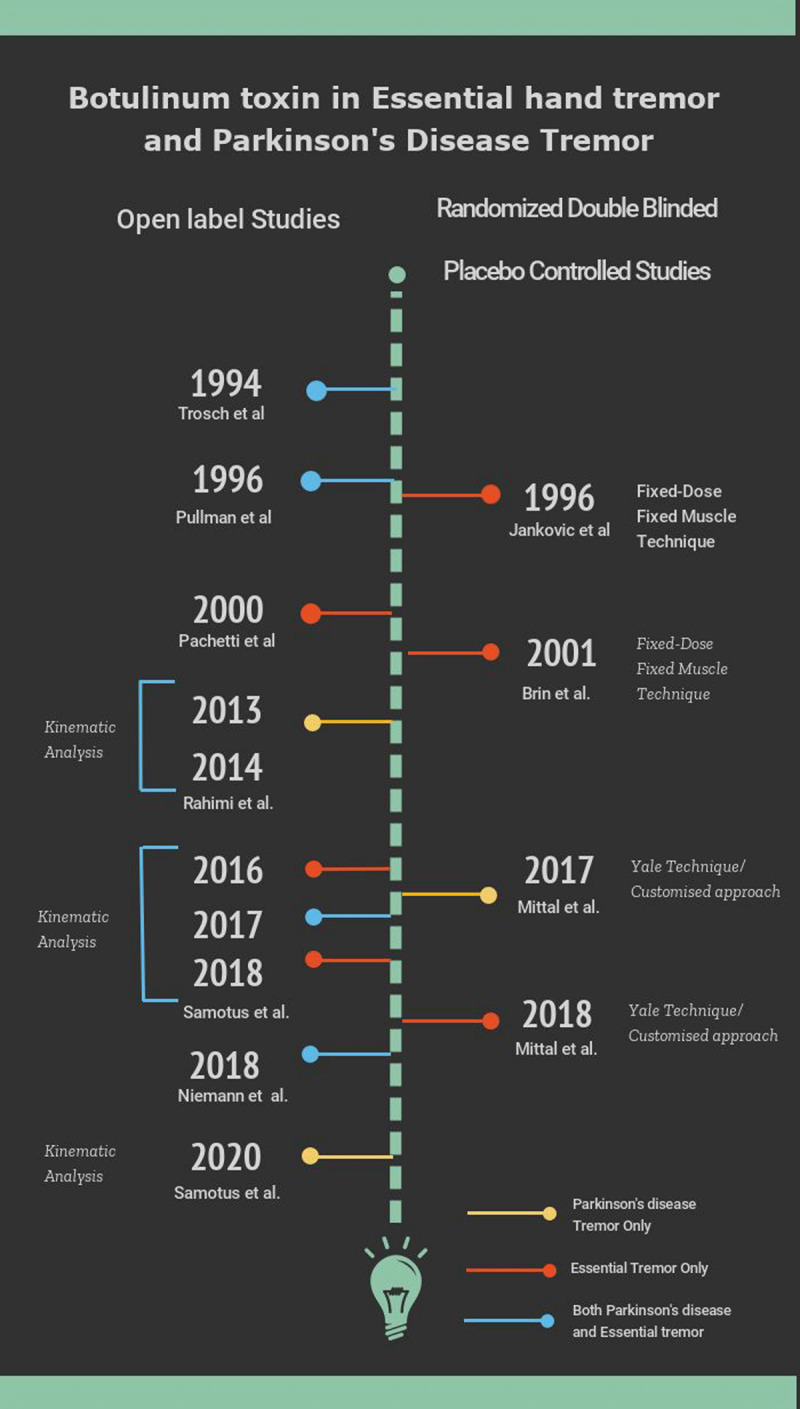
Timeline of the Botulinum toxin in Essential Tremor and Parkinson’s disease tremor.

**Table 1:** Class of clinical trials and definition of efficacy level endorsed by the Assessment and Guidance Committee of the American Academy of Neurology*.


Study Class
Class I: A randomized clinical trial of the invention of interest with masked or objective outcome assessment, in a representative population. Relevant baseline characteristics are presented substantially equivalent among treatment groups or there is appropriate statistical adjustment for differences.
The following are also required: a. concealed allocation. b. primary outcome(s)clearly defined. c. exclusion/inclusion criteria clearly defined. d. adequate accounting for dropouts (with at least 80% of enrolled subjects completing the study) and cross- overs with numbers sufficiently low to have minimal potential for bias.
Class II: A randomized controlled clinical trial of the intervention of interest in a representative population with masked or objective outcome assessment that lacks one criteria a-d above or a prospective matched cohort study with masked or objective outcome assessment in a representative population that meets b-d above.
Class III: All other controlled trials (including well-defined natural history controls or patients serving as own controls) in a representative population, where outcome is independently assessed, or independently derived by objective outcome measurement.
Class IV: Studies not meeting Class I, II or III criteria including consensus or expert opinion.
Level of Evidence:
Level A (efficacy established, recommended or not recommended), required two class I or one class I and two class II studies.
Level B (probably effective or probably not effective), required one class I or two class II studies
Level C (possible effective or not effective), requires one class II study
Level U (efficacy undetermined): due to contradictory results or lack of quality studies.


Reproduced from references 16 and 17, excluding the criteria for non-inferiority clinical trials.

In a double-blind, placebo-controlled study, Jankovic et al [13] administered onabotulinumtoxinA or saline to 25 individuals with ET. Tremor was scored according to a functional grading scale with scores ranging from 0 to 4, indicating no tremor to severe tremor (>4 cm in amplitude). Exclusion criteria consisted of patients younger than 21 years of age and those who had other causes for tremor, coexisting tremor conditions, previous treatment and a functional score of more than 2. Injections were administered into the flexor carpi radialis (FCR), flexor carpi ulnaris (FCU), extensor carpi radialis (ECR) and extensor carpi ulnaris (ECU). The dosage included 15 units into each of the two former muscles and 10 units into each of the two latter muscles of the dominant hand, based on anatomic localization. A total of 50 units of the toxin were initially used and an additional 100 units were reinjected to patients who did not respond adequately to the first dose after several weeks. BoNT and saline groups were compared after one month. Following initial injections, improvement was observed in 75% of the onabotulinumtoxinA-treated patients versus 25% of the control group. A statistically significant difference in the percentage of patients showing improvement was observed between the control and toxin groups. However, the positive response in functional rating scales was not significant. Improvements were largely found in postural tremor. Adverse events included mild/moderate weakness of the hands (50% of patients) and fingers (42% of patients). This trial was the first double-blind placebo-controlled published study on BoNT treatment of individuals with ET.

The second investigation of this type was conducted by Brin et al [14], who injected the same four muscles of 130 patients with a total of either 50 or 100 units of onabotulinumtoxinA. The subjects were followed for a maximum of 16 weeks with scheduled visits at 1, 2, 4, 6, 8, 12 and 16 weeks. Each flexor and extensor muscle of the forearm was respectively injected with 15 and 10 units in the low-dose group and 30 and 20 units in the high-dose group. Postural tremor improved significantly from the 4^th^ to 16^th^ week of injection, while kinetic tremor improvement was noted only on week 6, post-injection. Side effects were more prevalent in the high-dose group with 70% of the patients experiencing hand and finger weakness, as opposed to 30% of the participants in the low-dose group. Both studies concluded that these adverse events were essential shortcomings of BoNT application in ET treatment. To overcome this crucial issue, avoiding injections into the extensor muscles responsible for weakness, in addition to replacement of fixed injection patterns with customized administrations have been proposed by Jankovic et al [13].

In a randomized, placebo-controlled, double-blind trial at Yale University, a customized approach to evaluate the effectiveness of incobotulinumtoxinA in the management of ET was employed [16]. The study used a cross-over design by which 28 subjects crossed over to the other group (toxin to placebo or vice versa), 4 months after the initial treatment. It was hypothesized that injection of muscles of the forearm demonstrating electromyographic (EMG) tremor activity could collectively increase the effectiveness of BoNT therapy.

Selection of muscles for injection: The authors screened 8 forearm muscles with EMG that in their experience with ET and PD tremor, were often active and only active muscles were injected. These were flexor carpi ulnaris (FCU), flexor carpi radialis (FCR), extensor carpi ulnaris (ECU), extensor carpi radialis (ECR), extensor carpi ulnaris (ECU), flexor digitorum superficialis (FDS) and flexor digitorum profundus (FDP), pronator teres and supinator muscles. Injections were performed through a hand-held EMG. device which assessed the rhythmic sound developed by muscular tremor. IncobotulinumtoxinA was administered only to those muscles which produced a distinct typical sound when tremor occurred. The dose for the treatment of wrist flexors and extensors was substantially lower that that of onabotulinumtoxinA applied in the former studies (10 units per each flexor and 2.5 unit per each extensor) [13,14]. Since the units of these two toxins are roughly comparable, comparisons would be valid. An additional measure in the study was the administration of a predetermined 20 units BoNT-A into the bicep and tricep muscles. This was based on the fact that proximal muscles are frequently associated with ET. All participants also received injections in the hand lumbrical muscles responsible for metacarpophalangeal joint movement in tremor. The mean number of injected muscles/patient and the mean total injected dose were 9 muscles and 100 units, respectively. In the study the National Institute of Health Collaborative Genetic Criteria (NIHCGC) tremor score, Fahn Tolosa Marin (FTM) scale, an ergometer and the Patient Global Impression of Change (PGIC) was utilized to document the severity of tremor (0 to 4 scale), writing and spiral drawing, hand strength and patient perception of tremor improvement (0–7 scale), respectively. The latter was measured at baseline, 4 weeks after the initial injection and every 4 weeks afterwards. All other assessments were evaluated at baseline and 4- and 6-weeks post-injection, which continued every 8 weeks until the end of the 32-week study period. For both postural and kinetic tremor, the NIHCGC, FTM and PGIC scores of the toxin-injected patients showed significant improvement on weeks 4 and 6, compared to the controls (P < 0.05). Six (20%) subjects showed adverse events which were limited to slight weakness in the hand, mainly determined through an ergometer. Moderate to severe weakness developed in the wrist extensors of only one (6.6%) participant.

Using the same methodology, another blinded and placebo-controlled, crossover trial at Yale was completed where 30 Parkinson tremor patients were injected with a incobotulinumtoxinA [15]. The protocol, including the screening method (EMG) and injection design were identical to the one applied for the ET patients [16]. The Parkinson Disease Quality of Life Scale (PDQLS) and Unified Parkinson’s Disease Rating Scale (UPDRS) were added to the previous assessment methods. Results indicated a significant enhancement (P < 0.05) of the NIHCGC, FTM and PGIC scores in the toxin group compared to the placebo group, on weeks 4 and 6. Similarly, there was an improvement in UPDRS section 20 and section 16 scores (ranges: 0–4) at the same time points, specifying changes in resting tremor (P < 0.001) and symptomatic complaint of tremor (P < 0.05), respectively. Section 21 of the UPDRS (range: 0–4) is reflective of action/postural tremor which also showed significant improvement, but only on week 8 (P = 0.01). Despite the superior PDQLS results in the toxin group, the difference did not reach statistical significance. Ergometric assessment demonstrated minor weakness in 37% of the participants and moderate weakness of the hand in 2 (13.5%) patients, one of which was reported only in the finger extensors. This was the first and the only study conducted as a double blind, placebo-controlled trial to determine the effectiveness and adverse events of BoNT injections in PD tremor.

The kinematic tremor analysis and standardized assessment was first reported by Rahimi et al [20] and Samotus et al [21] to improve the accuracy of muscle selection and dosing for BoNT therapy in ET and PD patients. In this method, four motion sensors are placed on the whole arm to measure angular tremor amplitude at each joint which were then analyzed into directional elements associated with various muscle groups. Samotus et al [17] applied this assessment procedure in a prospective open-label study and evaluated tremor activity in forearm (7 muscles) and arm (6 muscles) of 52 participants, 24 of which suffered from ET and the rest had tremor due to Parkinson disease. IncobotulinumtoxinA (70 to 300 units/session) was administered into active muscles every 16 weeks starting at baseline, clinical determination of the treatment pattern was aided by the objective analysis of each individual’s tremor characteristics. The patients were followed every 6 weeks after each of the injections, adding up to a total of 96 weeks of follow-up. Therapeutic effectiveness was measured using the FTM scale, quality of life (QoL) questionnaire and manual muscle testing. Side effects were assessed through maximum grip strength and perceived muscle weakness. Tremor amplitude showed a reduction of 70%–76% for both diseases throughout the study period (96 weeks). The QoL demonstrated significant improvement in the ET patients (p < 0.05), but not immediate in individuals with PD. Functional interference induced by tremor significantly improved to the end of the study period in both ET and PD patients. Participation was withdrawn by 8% and 14% of the ET and PD patients respectively, due to bothersome hand and finger weakness. Correspondingly another 8% and 11% of the patients withdrew as a result of functional ineffectiveness. The conclusion of this study indicated significantly improved upper limb functionality in BoNT-injected patients who had whole arm tremor and were examined with the computer-assisted tremor method to aid in the planning of the initial treatment pattern. This procedure proved to eliminate subjectivity in identification and enabled customization of muscle patterns for BoNT therapy.

Computer-assisted tremor analysis was employed in another open label study to evaluate the effectiveness and safety of bilateral BoNT-A injections in 31 patients with ET [22]. In each arm, the muscles of the wrist, elbow and shoulder were assessed with this procedure and incobotulinumtoxinA injections were individualized for all participants. Tremor severity and QoL improved significantly (P < 0.005), based on the FTM scale and QoL questionnaire, between 6 to 30 weeks after injection. It was concluded that the use of a personalized strategy for bilateral injections provided effective tremor control in patients with ET and could eliminate the variability underlying clinical evaluation and accelerate the regain of arm function. Similar strategy was employed in another recent study to evaluate the effectiveness and safety of BoNT-A in 47 PD participants who received 4 serial BoNT-A treatments with follow-ups at 6 weeks post-treatment over 42 weeks [23]. There was significant improvement in tremor amplitude and UPDRS tremor score (p < –.05) with improvement in the QoL. In this study, almost half of the patients were not on levodopa therapy, the authors suggest that BoNT-A could be considered as first line treatment in tremor-dominant PD patients.

In a retrospective review, Niemann and Jankovic [24] reported their longitudinal 8-year experience with onabotulinumtoxinA in the management of hand tremor resulting from ET (N = 53), dystonic tremor (N = 31), PD (N = 6) and cerebellar outflow tremor (N = 1). The basis for muscle selection was palpation and surface anatomy in the majority (94.5%) of participants. Most injections were administered into the FCR and FCU muscles. Pronator teres, FDS, biceps and triceps received injections with less frequency. The mean follow-up was 2.5 years. Tremor improved significantly, even though a 21% dosage increase per limb was required to maintain the results. Adverse events in the form of hand and finger weakness were observed in 12% of the subjects, but its occurrence was not reported separately for ET and PD. Approximately 40% of the participants dropped out after the first session as a result of unwanted side-effects or no noticeable benefit. Using anatomical localization for selecting target muscles and avoiding wrist extensor injections, the authors declared onabotulinumtoxinA to be a relatively risk-free and successful treatment method for the management of patients with refractory hand tremor. While recognizing the limitations of retrospective data, they claimed this method to provide comparable results to that of using EMG (Yale protocol) or kinematic tremor analysis, without the extra cost, patient discomfort and time loss.

## Discussion and Conclusion

In order for a specific treatment to acquire “established efficacy”, the Assessment and Guidance Committee of the American Academy of Neurology has recommended the publication of either two class I studies or one class I along with two class II studies (Table 1). Accordingly, following the addition of the recent class I study on ET [16] to the two former class II trials [13,14], BoNT-A injection can be considered to have “established efficacy” in the management of ET patients. On the other hand, based on the single class I study on PD tremor conducted by the Yale group [15], BoNT-therapy could be regarded as “probably” effective for treatment of individuals with PD. Currently, experts in the field of movement disorders suggest BoNT injections to be used for ET and PD patients refractory to drug treatment and those who cannot be subjected to DBS or do not agree to have the procedure [24,25].

One of the issues with BoNT-therapy in ET and PD patients is weakness occurring in the hands and fingers. This side effect has been considerably overcome, following the introduction of new methods like the Yale protocol [15,16] and computerized kinematic tremor assessment [17,20,22]. The former applies EMG to screen 8–10 forearm muscles prior to injection and includes two proximal and lumbrical muscles to plan the treatment pattern. In the latter, multi-sensor kinematic technology is used on the whole arm to aid in determination of tremor muscle groups to customize the BoNT injection pattern in 7 forearm and 6 proximal muscles. A recent collaborative review, highlighted the advantages of using customized toxin administrations over fixed-dose-fixed-muscle injections for the treatment of tremor [26].

Localization of tremulous muscles based on palpation and surface anatomy has been suggested by the Baylor group when treating hand tremor [24]. In this method, a limited number of muscles, mostly FCU and FCR were injected, and it has been claimed to be more time- and cost-effective than the Yale technique and computerized kinematic tremor assessment. However, the severity of tremor patients in each study differed making the comparison baseline unequal. The retrospective study reported hand tremor patients that required forearm injections were treated with an average starting dose of 65U, and likely had only wrist treatment. In contrast, the EMG studies treated individuals with wrist and elbow tremor, and the kinematic studies included patients with severe tremor involving the whole arm with an average starting dose of 174U for PD and 169U for ET [17]. More specifically, the kinematic method did not exclude any severe tremor patients from their study and included individuals that were candidates for neurostimulation but opted to explore BoNT-A study first.

Both the Yale and kinematic studies achieved tremor suppression and the regaining of arm function using higher BoNT-A doses in more severe patients and had similarly low side-effect profiles and discontinuation as compared to the Baylor group’s hand tremor study. From the time and cost perspective, there is significant value and opportunity to leverage technology to address the treatment gap tremor patients face. Currently, the treatment plan is to offer oral medication to manage the patient’s tremor symptoms [6,7]; however, if the medication cannot be tolerated or when their hand and arm tremor symptoms worsen to further impact their function then neurostimulation or ablation therapy is offered as the next option [5]. Both surgical options are resource intensive, high cost, and carry a degree of risk to the patient. The prospective studies which used of more extensive EMG screening (Yale method) or the kinematic technologies to plan the treatment pattern of BoNT-A therapy have demonstrated significant value in achieving tremor suppression, quality of life, and the return of arm function in activities of daily living and at work.

From a practice perspective, the use of palpation and knowledge of surface anatomy is crucial for the localization of some forearm muscles during the needle insertion into the muscle belly for delivery BoNT-A therapy. However, this technique will be challenging to implement for muscles that are localized deeper in the arm. Likewise, palpation and visual inspection to guide clinical decision for BoNT-A pattern and dosing to treat hand tremor is highly subjective and dependent on the clinical expertise of the injector and the feasibility for treating whole arm tremor using palpation technique has not been studied. Whereas the use of planning technology can be applied to all severities of hand and arm tremor, and provides objective analyzed data, offering an easier standardized approach. When examining the use of the Yale technique, the EMG probing of each muscle in the arm can provide clinical insight on which muscles and groups of muscles are contribute the tremor at the wrist, elbow, and shoulder joint. Furthermore, EMG analysis and clinical interpretation of the signal aids in the determination of dose per muscle to create the treatment pattern. One limitation with the EMG technique is the possible discomfort associated with needle insertion into the muscle, and multiple needles cannot be inserted into all the muscles at the same time to allow for synchronized recording.

The kinematic planning method offers similar insight to the EMG technique, where it can generate objective analysis on the sources of tremor in the arm and which muscles groups at the wrist, elbow, and shoulder are causing the tremor and the severity for each directional element. Although the kinematic technology can simultaneously record and analyze the tremor from the whole arm, it is not able to provide the same fidelity of information such as needle EMG on individual muscle activities. Nonetheless, the use of motion sensors does allow the patient to perform multiple arm tasks and ADLs to enable the shared decision between physician and patient on which task and goals they want the BoNT-A treatment to target. Both methods (Yale EMG method and Kinematic method) have produced significantly less weakness compared to previously reported methods. Only a comparator study can answer that which method is superior to the other, considering efficacy, cost of the instruments, analysis/interpretation fee, the cost-benefit of having better tremor suppression sooner or avoiding surgery, and the time required for each method to provide satisfactory results. The role of other powerful techniques such as sonography to define forearm muscles participating in ET and PD tremor and guide BoNT injections also need to be investigated by controlled studies. The correct dose per muscle and selection of proximal muscles for injection still needs to be further investigated in both Yale and kinematic method. For instance, in Yale method, the dose of 20 units for biceps and triceps muscles might have been too small to be effective in the selected participants. In kinematic method, some of the patients received much higher dose of BoNT in biceps and triceps muscles based on the onjective severity of the tremor at the elbow joint.

Ultimately to best clarify whether the outcome of the subjective palpation method could compete with the technology-based techniques and procedures in terms of efficacy and negligible side-effects for all severities of hand and arm tremor requires additional prospective double-blind, placebo-controlled studies.

## References

[1] Louis ED. Diagnosis and Management of Tremor. Continuum (Minneap Minn). 2016 8; 22(4 Movement Disorders): 1143–58. DOI: 10.1212/CON.0000000000000346 27495202

[2] Bhatia KP, Bain P, Bajaj N, Elble RJ, Hallett M, Louis ED, et al. Consensus Statement on the classification of tremors, from the task force on tremor of the International Parkinson and Movement Disorder Society. Movement Disorders. 2018 1; 33(1): 75–87. DOI: 10.1002/mds.27121 29193359 PMC6530552

[3] Poewe W, Seppi K, Tanner CM, Halliday GM, Brundin P, Volkmann J, et al. Parkinson disease. Nat Rev Dis Prim [Internet]. 2017; 3(1): 17013 DOI: 10.1038/nrdp.2017.13 28332488

[4] Lyons MK. Deep Brain Stimulation: Current and Future Clinical Applications. Mayo Clin Proc [Internet]. 86(7): 662–72. DOI: 10.4065/mcp.2011.0045 21646303 PMC3127561

[5] Haubenberger D, Hallett M. Essential Tremor. N Engl J Med. 2018 5; 378(19): 1802–10. DOI: 10.1056/NEJMcp1707928 29742376

[6] Louis ED, Rohl B, Rice C. Defining the Treatment Gap: What Essential Tremor Patients Want That They Are Not Getting. Tremor Other Hyperkinet Mov (N Y). 2015 8 14; 5: 331 DOI: 10.5334/tohm.239 26317044 PMC4548969

[7] Fasano A, Deuschl G. Therapeutic advances in tremor. Mov Disord. 2015 9; 30(11): 1557–65. DOI: 10.1002/mds.26383 26293405

[8] Baizabal-Carvallo JF, Kagnoff MN, Jimenez-Shahed J, Fekete R, Jankovic J. The safety and efficacy of thalamic deep brain stimulation in essential tremor: 10 years and beyond. J Neurol Neurosurg Psychiatry. 2014 5; 85(5): 567–72. DOI: 10.1136/jnnp-2013-304943 24096713

[9] Elias WJ, Lipsman N, Ondo WG, Ghanouni P, Kim YG, Lee W, et al. A Randomized Trial of Focused Ultrasound Thalamotomy for Essential Tremor. N Engl J Med. 2016 8; 375(8): 730–9. 27557301 10.1056/NEJMoa1600159

[10] Rohani M, Fasano A. Focused Ultrasound for Essential Tremor: Review of the Evidence and Discussion of Current Hurdles. Tremor Other Hyperkinet Mov (N Y) [Internet]. 2017 5 5; 7: 462 Available from: https://pubmed.ncbi.nlm.nih.gov/28503363 DOI: 10.5334/tohm.378 28503363 PMC5425801

[11] Safarpour Y, Jabbari B. Botulinum Toxin Treatment of Movement Disorders. Curr Treat Options Neurol. 2018 2; 20(2): 4 DOI: 10.1007/s11940-018-0488-3 29478149

[12] Pirazzini M, Rossetto O, Eleopra R, Montecucco C. Botulinum Neurotoxins: Biology, Pharmacology, and Toxicology. Pharmacol Rev. 2017 4; 69(2): 200–35. DOI: 10.1124/pr.116.012658 28356439 PMC5394922

[13] Jankovic J, Schwartz K, Clemence W, Aswad A, Mordaunt J. A randomized, double-blind, placebo-controlled study to evaluate botulinum toxin type A in essential hand tremor. Mov Disord [Internet]. 1996/5/01 1996; 11(3): 250–6. DOI: 10.1016/j.mayocp.2017.06.010 8723140

[14] Brin MF, Lyons KE, Doucette J, Adler CH, Caviness JN, Comella CL, et al. A randomized, double masked, controlled trial of botulinum toxin type A in essential hand tremor. Neurology [Internet]. 2001/6/13 2001; 56(11): 1523–8. DOI: 10.1212/WNL.56.11.1523 11402109

[15] Mittal SO, Machado D, Richardson D, Dubey D, Jabbari B. Botulinum Toxin in Parkinson Disease Tremor: A Randomized, Double-Blind, Placebo-Controlled Study With a Customized Injection Approach. Mayo Clin Proc. 2017/8/10 2017; 92(9): 1359–67. DOI: 10.1016/j.mayocp.2017.06.010 28789780

[16] Mittal SO, Machado D, Richardson D, Dubey D, Jabbari B. Botulinum toxin in essential hand tremor – A randomized double-blind placebo-controlled study with customized injection approach. Parkinsonism Relat Disord. 2018 11; 56: 65–69. DOI: 10.1016/j.parkreldis.2018.06.019 29929813

[17] Samotus O, Lee J, Jog M. Long-term tremor therapy for Parkinson and essential tremor with sensor-guided botulinum toxin type A injections. PLoS One. 2017; 12(6): e0178670 DOI: 10.1371/journal.pone.0178670 28586370 PMC5460844

[18] French J, Gronseth G. Lost in a jungle of evidence: we need a compass. Neurology. 2008 11; 71(20): 1634–8. DOI: 10.1212/01.wnl.0000336533.19610.1b 19001254

[19] Gronseth G, French J. Practice parameters and technology assessments: what they are, what they are not, and why you should care. Neurology. 2008 11; 71(20): 1639–43. DOI: 10.1212/01.wnl.0000336535.27773.c0 19001255

[20] Rahimi F, Samotus O, Lee J, Jog M. Effective Management of Upper Limb Parkinsonian Tremor by IncobotulinumtoxinA Injections Using Sensor-based Biomechanical Patterns. Tremor Other Hyperkinet Mov (N Y) [Internet]. 2015/11/14 2015; 5: 348 DOI: 10.5334/tohm.240 26566459 PMC4636031

[21] Samotus O, Rahimi F, Lee J, Jog M. Functional Ability Improved in Essential Tremor by IncobotulinumtoxinA Injections Using Kinematically Determined Biomechanical Patterns – A New Future. PLoS One [Internet]. 2016/4/23 2016; 11(4): e0153739 DOI: 10.1371/journal.pone.0153739 27101283 PMC4839603

[22] Samotus O, Lee J, Jog M. Personalized Bilateral Upper Limb Essential Tremor Therapy with Botulinum Toxin Using Kinematics. Toxins (Basel). 2019 2; 11(2). DOI: 10.3390/toxins11020125 PMC6409675 30791440

[23] Samotus O, Lee J, Jog M. Standardized algorithm for muscle selection and dosing of botulinum toxin for Parkinson tremor using kinematic analysis. Ther Adv Neurol Disord. 2020; 13: 1756286420954083 DOI: 10.1177/1756286420954083 33014139 PMC7517980

[24] Niemann N, Jankovic J. Botulinum Toxin for the Treatment of Hand Tremor. Toxins (Basel). 2018 7; 10(7). DOI: 10.3390/toxins10070299 PMC6070882 30029483

[25] Zakin E, Simpson D. Botulinum Toxin in Management of Limb Tremor. Toxins (Basel). 2017 11; 9(11). DOI: 10.3390/toxins9110365 PMC5705980 29125566

[26] Mittal SO, Lenka A, Jankovic J. Botulinum toxin for the treatment of tremor. Park Relat Disord. 2019 6; 63: 31–41. DOI: 10.1016/j.parkreldis.2019.01.023 30709779

